# A multicenter real‐life study to determine the efficacy of corticosteroids and olfactory training in improving persistent COVID‐19‐related olfactory dysfunction

**DOI:** 10.1002/lio2.989

**Published:** 2022-12-02

**Authors:** Alfonso Luca Pendolino, Giancarlo Ottaviano, Juman Nijim, Bruno Scarpa, Giulia De Lucia, Cecilia Berro, Piero Nicolai, Peter J. Andrews

**Affiliations:** ^1^ Department of ENT Royal National ENT & Eastman Dental Hospitals London UK; ^2^ Ear Institute University College London London UK; ^3^ Department of Neurosciences, Otolaryngology Section University of Padova Padova Italy; ^4^ University College London Medical School London UK; ^5^ Department of Statistical Sciences and Department of Mathematics Tullio Levi‐Civita University of Padova Padova Italy

**Keywords:** corticosteroids, COVID‐19, olfaction, olfactory training, smell

## Abstract

**Background:**

No definitive treatment exists to effectively restore function in patients with persistent post‐infectious olfactory dysfunction (OD). Corticosteroids have been considered as a therapeutic option in post‐infectious OD but their benefit in COVID‐19‐related OD remains unexplored. We aim to determine the role of the combination of corticosteroids plus olfactory training (OT) in improving persistent COVID‐19‐related OD.

**Methods:**

A multicenter real‐life cohort study was conducted between December 2020 and April 2022 on patients with reported COVID‐19‐related OD. Only patients with confirmed OD at Sniffin' Sticks (S'S) and those who attended their 6‐month follow‐up were included. Patients were started on a combined treatment of corticosteroids and OT. Patients refusing corticosteroids or not doing any treatment formed the control groups. Visual analogue scale (VAS) for sense of smell and SNOT‐22 were used to assess patients reported symptoms.

**Results:**

Sixty‐seven subjects with reported COVID‐19‐related OD were initially seen. Normosmic patients at S'S (*n* = 14) and those not attending their follow‐up (*n* = 9) were excluded. Of the 44 patients included in the analysis, 19 patients had the combined treatment (group A), 16 patients refused to take corticosteroids and did the OT alone (group B) whereas 9 patients did not do any treatment (group C). An improvement of threshold + discrimination + identification (TDI) score (*p* = .01) and VAS for smell (*p* = .01) was found in group A whereas only the TDI score improved in group B (*p* = .04). Presence of comorbidities, age, sex (male), and length of OD negatively influenced olfactory recovery.

**Conclusions:**

Our study confirms the importance of OT in long‐term OD suggesting that the addition of corticosteroids may give a benefit in terms of patient's perceived olfaction.

**Level of Evidence:**

2b

## Abstract 2

No definitive treatment exists to effectively restore function in patients with persistent post‐infectious olfactory dysfunction(OD). Corticosteroids have been considered as a therapeutic option in post‐infectious OD but their benefit in COVID‐19‐related OD remains unexplored. Our study confirms the importance of OT in long‐term OD suggesting that the addition of corticosteroids may give a benefit in term of patient's perceived olfaction.

## 1 INTRODUCTION

Olfactory dysfunction (OD) represents a highly prevalent symptom in patients infected by the severe acute respiratory syndrome coronavirus 2 (SARS‐CoV‐2) with up to 85% of mild‐to‐moderate coronavirus disease 2019 (COVID‐19) cases developing loss of sense of smell.^1^, ^2^ Spontaneous recovery rate of olfaction is very high within the first month following infection (recovery rate 94.6%) and it becomes 85.7% at 6 months^3^ and 93% at 12 months.^4^ Persistent post‐infectious OD (PIOD) has been recognized as a “long‐COVID” symptom, defined as a persistent symptom in individuals who recovered from COVID‐19^5^ and, unfortunately, no definitive treatments exist to effectively restore function. European guidelines recommend olfactory training (OT) for a minimum of 3 months to maximize the chance of smell improvement.^6^ Nonetheless, OT remains ineffective in 50%–85% of subjects^7^, ^8^, ^9^ with up to 29% of PIOD cases not improving even after long‐term OT (14 months).^10^

Topical and systemic corticosteroids have been considered as a therapeutic option in PIOD but their benefits for non‐sinonasal‐related OD remain controversial. A systematic review published in 2019^11^ suggested that systemic corticosteroids could improve olfactory loss in PIOD (Level 4), whereas a more recent one^12^ concluded that systemic or topical corticosteroids remain “optional” due to the lack of high‐quality studies. The rationale behind the use of corticosteroids to treat PIOD relies on its capacity to reduce a subclinical inflammation which may persist in the nose after an otherwise resolved upper respiratory tract infection. On the other hand, corticosteroids could play a role in the regeneration of the olfactory epithelium of PIOD patients, as already shown in animal models.^13^, ^14^ Studies focusing on corticosteroids as treatment of PIOD did not clarify which formulation, dose and route of administration is better in improving sense of smell and if this is more effective if combined with OT. Another question remains on whether there is a time limit from OD onset at which treatment should be started in order to observe a benefit. Ultimately, in the lack of clear evidence‐based guidelines the choice is left to doctor's preferences. To date, most of the authors seem to agree that corticosteroids may have a role when started close to OD onset^15^; however, whether this could have a role in persistent OD remains partially unexplored.

In this study, we aim to investigate the role of the combination of corticosteroids plus OT in improving persistent COVID‐19‐related OD in a cohort of subjects with a history of smell loss longer than 7 months. Patients refusing to take corticosteroids and doing OT alone and those not doing any treatment were used as internal controls.

## 2 MATERIALS AND METHODS

### 2.1 Study design

A multicenter real‐life cohort study was conducted to assess the efficacy and safety of corticosteroids in combination with OT in the treatment of persistent OD in patients with a history of mild‐to‐moderate COVID‐19. The study was approved by the Hospital Research Ethic Committees (REC ref 14/SC/1180) and was conducted in accordance with the Declaration of Helsinki.

### 2.2 Participants' characteristics

Patients with a reported OD that occurred following a laboratory‐confirmed SARS‐CoV‐2 infection referred to our smell clinics at the University College London Hospitals (London, United Kingdom) and the University Hospital of Padua (Padua, Italy) were selected. All participants provided full informed consent prior to their inclusion in the study. Data were collected on demographics, subjective characteristics of OD at onset, smoking status, comorbidities, and medications taken (Table 1). Patients with a chronic or recent short‐term oral steroid use, pregnancy, pre‐existing history of OD, non‐COVID‐19‐related OD, or other pathologies known to affect olfaction (i.e., head and neck tumors, chronic rhinosinusitis [CRS], head trauma, radio/chemotherapy of the craniofacial region, psychiatric or neurological disease) were not included in the study.

**TABLE 1:** General characteristics of the whole population of dysosmic patients and according to type of treatment

	Patients with OD (*n* = 44)	Group A_S+OT_ (*n* = 19)	Group B_OT_ (*n* = 16)	Group C_None_ (*n* = 9)	*p*‐value
Age, median [P25‐P75], years	40.5 [30.5–53.3]	47.0 [31.0–54.0]	50.0 [33.0–57.0]	32.0 [28.0–35.0]	.03*
Sex, no (%)					
Female	28 (63.6%)	11 (57.9%)	11 (68.8%)	6 (66.7%)	.78
Male	16 (36.4%)	8 (42.1%)	5 (31.2%)	3 (33.3%)	
Comorbidities, no (%)					
Diabetes	1 (2.3%)	1 (5.3%)	0 (0.0%)	0 (0.0%)	.36
Hypertension	4 (9.1%)	1 (5.3%)	3 (18.8%)	0 (0.0%)	
Hyperlipidemia	3 (6.8%)	1 (5.3%)	2 (12.5%)	0 (0.0%)	
Hypothyroidism	1 (2.3%)	1 (5.3%)	0 (0.0%)	0 (0.0%)	
Allergic rhinitis	1 (2.3%)	1 (5.3%)	0 (0.0%)	0 (0.0%)	
Smoking, no (%)	5 (11.4%)	1 (5.3%)	2 (12.5%)	2 (22.2%)	.41
Medications, no (%)					
None	35 (79.5%)	13 (68.4%)	13 (81.3%)	9 (100%)	.28
Yes	9 (20.5%)	6 (31.6%)	3 (18.7%)	0 (0.0%)	
α‐blockers	0 (0.0%)	0 (0.0%)	0 (0.0%)	0 (0.0%)	
Sartans	1 (11.1%)	0 (0.0%)	1 (25.0%)	0 (0.0%)	
Dicumarolics	0 (0.0%)	0 (0.0%)	0 (0.0%)	0 (0.0%)	
Antiplatelet drugs	3 (33.3%)	1 (16.7%)	2 (50.0%)	0 (0.0%)	
Biguanides	0 (0.0%)	0 (0.0%)	0 (0.0%)	0 (0.0%)	
Antidepressants	1 (11.1%)	1 (16.7%)	0 (0.0%)	0 (0.0%)	
Others	8 (88.9%)	6 (100%)	2 (50.0%)	0 (0.0%)	
Interval for smell loss onset, median [P25‐P75], days	1.0 [0.0–4.3]	2.0 [0.0–7.0]	0.5 [0.0–3.0]	1.0 [0.0–5.0]	.96
Length of OD [P25‐P75], days	224.0 [136.0–383.8]	214.0 [165.5–352.5]	226.5 [126.3–418.0]	235.0 [191.0–383.0]	.94
Reported level of smell at infection, no (%)					
Anosmia	36 (81.8%)	17 (89.5%)	11 (68.8%)	8 (88.9%)	.24
Hyposmia	8 (18.2%)	2 (10.5%)	5 (31.2%)	1 (11.1%)	
Previous treatments, no (%)					
Olfactory training	27 (61.4%)	11 (57.9%)	13 (81.3%)	3 (33.3%)	.06
Oral steroid	2 (4.5%)	2 (10.5%)	0 (0.0%)	0 (0.0%)	.25
Topical steroid (drops)	2 (4.5%)	2 (10.5%)	0 (0.0%)	0 (0.0%)	.13
Topical steroid (spray)	8 (18.2%)	6 (31.6%)	1 (6.3%)	1 (11.1%)	.05
Multivitamins	20 (45.5%)	7 (36.8%)	11 (68.8%)	2 (22.2%)	.31

Abbreviations: OD, olfactory dysfunction; Others: anxiety, migraine, prolapsed discs, epilepsy, temporal arteritis, sleep problem, osteoporosis, asthma, dermatitis, IBS, eosinophilia, psoriasis, restless leg syndrome, CAD, osteoarthritis, VITD deficiency, bladder incontinence.

* Significant *p*‐values. Level of significance *p* < .05.

### 2.3 First assessment (T_0_ ) and evaluation of olfactory function

On the first visit, a fully detailed medical history was obtained. Participants were asked to report any medications they used. Factors such as duration of olfactory loss and presence of parosmia, described as the occurrence of distorted olfaction when smelling odor, were also explored. All patients underwent nasal endoscopy to exclude signs of CRS—nasal polyps, nasal discharge, and signs of rhinitis—or an obstruction/inflammation of the olfactory clefts. An MRI of the head was arranged for all patients to study the olfactory system and exclude any central causes of OD. Olfaction was evaluated using Sniffin' Sticks (S'S)—extended set (Burghart, Medisense) to obtain the odor threshold (T), discrimination (D), and identification (I) scores. Normosmia was attributed where TDI score (the sum of T, D, and I individual scores) was ≥30.75, hyposmia where TDI was >16, but <30.75, and functional anosmia if TDI ≤ 16.^16^ Self‐assessment of olfaction was performed using a visual analogue scale (VAS—0 represents “sense of smell absent” and 10 “sense of smell not affected”)^17^ whereas sinonasal symptoms were evaluated using the Sino‐Nasal Outcomes Test‐22 (SNOT‐22).^18^

### 2.4 Treatment and further follow‐up (T1)

Patients with no OD at S'S (TDI ≥ 30.75) were discharged back to their general practitioner (GP). Conversely, patients with a confirmed OD (TDI < 30.75) were offered a steroid treatment consisting of a 2‐week course of oral corticosteroids (Prednisolone 40 mg/daily for 5 days, then tapered down over 9 days) followed by intranasal corticosteroids drops for 2 weeks (Betamethasone 0.1%, 2 drops/nostril bidaily) administered in the Kaiteki position.^19^ Specific consent to start the previously mentioned treatment was sought from all patients before giving any related prescription. They were also asked to start OT, as previously described,^6^ until further follow‐up irrespective of whether they had done or not it before. Patients with contraindications to corticosteroids^20^ or refusing to take them were asked to start OT. A further follow‐up at 6 months was arranged for all patients and patient‐reported outcome measures (PROMs) and S'S were repeated on that occasion. Treatment adherence was checked at follow‐up by requesting specific questions about treatment (i.e., modalities of topical steroid drops administration, length of time allowed for OT, and strict adherence to instructions provided). At follow‐up, patients who did not do any treatment during the study period were kept in the analysis and formed an additional control group.

### 2.5 Statistical analysis

Quantitative variables were presented as median and interquartile range whereas qualitative variables were expressed as number of observations and percentage. Considering the Wilcoxon test, to obtain an increase in the TDI score of 5.5 points, which corresponds to the minimal clinically important difference (MCID),^21^ a power (1 − *β*) of 0.8 is obtained with *n* = 17 in each arm, whereas a sample size of *n* = 15 in each arm gives a power of 0.79, keeping a fix *α* (uncertainty level) at 5%. Comparisons of general characteristics and findings between groups were performed using the Kruskal‐Wallis test for quantitative variables and the Pearson chi‐square test for categorical variables. Differences between T_0_ and T_1_ were evaluated using the paired Wilcoxon test for quantitative variables whereas the chi‐square test was chosen for parosmia. Multiple linear regression with selection of variable based on Akaike's information criterion (backward stepwise) has also been performed to identify the effects of the available variables on the measurement changes at T_1_. *p*‐values have been calculated for all tests, and 5% was considered as the critical level of significance. All the analysis has been performed in R (R Core Team, 2021).

## 3 RESULTS

### 3.1 Breakdown of the population

**FIGURE 1:**
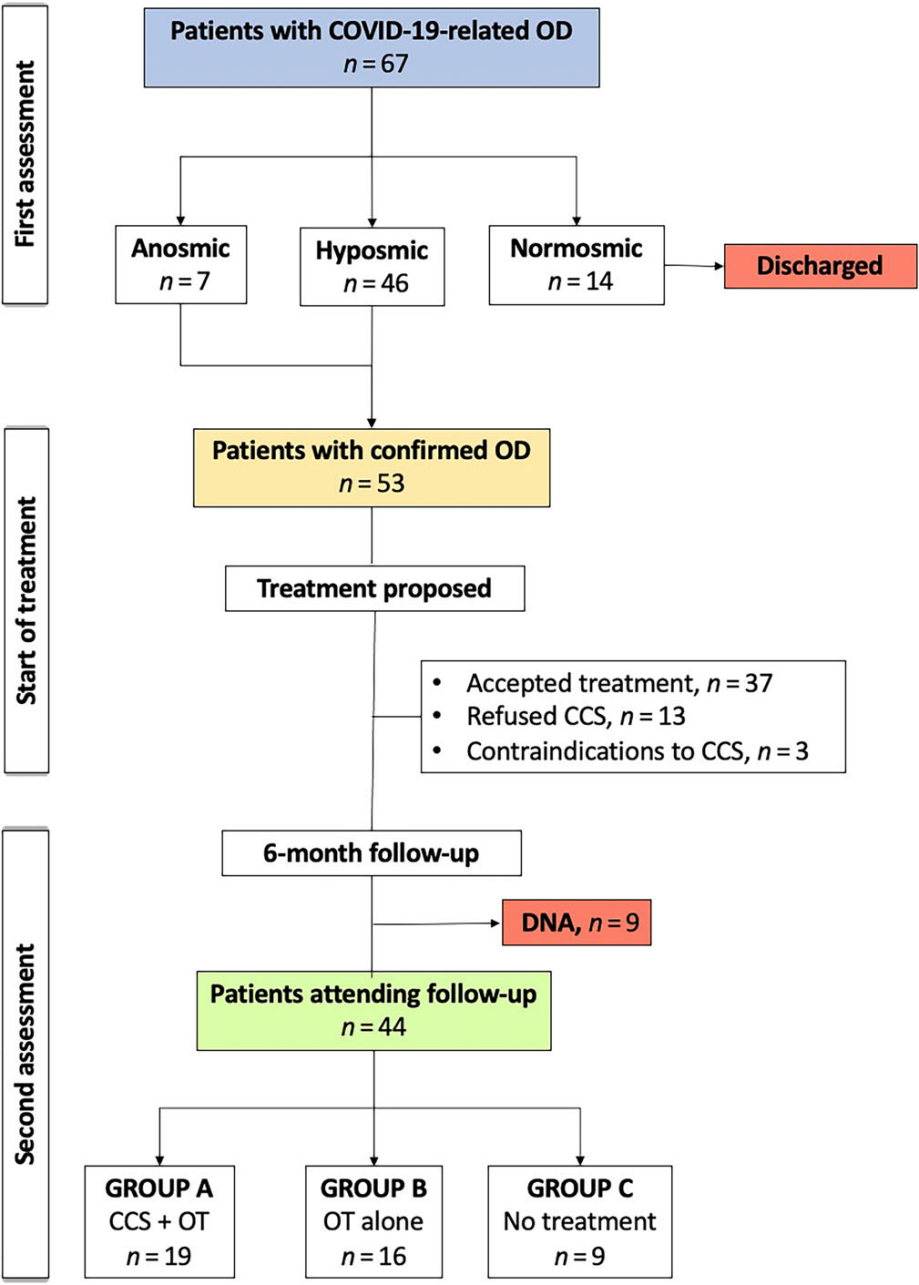
Flow chart of study population. CCS, corticosteroids; DNA, did not attend; OD, olfactory dysfunction; OT, olfactory training

Between December 2020 and April 2022, 67 patients with a reported COVID‐19‐related OD were seen at our smell clinics. All patients had a history of mild‐to‐moderate COVID‐19 and none of them required hospital admission. Of them, 14 patients were found to be normosmic at S'S and were discharged back to GP care. The remaining 53 subjects (7 anosmics) were advised to start the suggested treatment. Nine patients did not attend their 6‐month follow‐up leading to a total of 44 patients (28 female; 63.6%), with a median age of 40.5 years, who completed the study period and were considered for data analysis. Of them, 19 patients had the combined treatment (corticosteroids plus OT—group A), 16 patients refused to take corticosteroids and did the OT alone (group B) and 9 patients did not do any treatment despite medical recommendations (group C). Figure 1 shows the flow chart for the study population.

### 3.2 General characteristics of the population

Demographics, smoking status, comorbidities, and medications taken are reported in Table 1. All patients had a confirmed persistent COVID‐19‐related OD at S'S with a median length of OD of 224 days (calculated as number of days from the infection date to the day of first consultation). In most of the cases, this presented as a complete loss of sense of smell (36; 81.8%) and occurred at a median time of 1 day following infection. Most of the patients tried OT (27; 61.4%) or oral multivitamins (20; 45.5%) before coming for their first consultation. None of them received any course of oral steroid for their OD in the past. No side effects were reported after treatment with corticosteroids. Characteristics for each group of patients are reported in Table 1. Patients in group C were significantly younger (*p* = .03) but, apart from that, no other statistically significant differences were noted in terms of demographics and baseline clinical characteristics among the three groups (Table 1).

### 3.3 PROMs, olfactory measurements, and other investigations

Nasal endoscopy showed a clear olfactory cleft for all patients. MRI scan of the head was normal in all patients with no radiological sign of CRS or central causes of OD. PROMs scores (VAS and SNOT‐22), incidence of parosmia, threshold, discrimination, identification, and TDI scores at baseline (T_0_) and at follow‐up (T_1_) for each group of patients are reported in Table 2. Apart from a significant lower number of parosmics observed in group B at baseline (*p* = .01), no other significant differences were observed in the measurements either at baseline or at follow‐up in the three groups (Table 2).

**TABLE 2:** Measurements at baseline and follow‐up

	Patients with OD (*n* = 44)	Group A_S+OT_ (*n* = 19)	Group B_OT_ (*n* = 16)	Group C_None_ (*n* = 9)	*p*‐value_A‐B‐C_
Findings at first assessment (T_0_)					
Sniffin' Sticks, median [P25‐P75]					
Threshold	3.5 [1.0–5.5]	4.5 [1.0–5.3]	3.8 [1.0–5.5]	2.5 [2.3–6.0]	.95
Discrimination	10.0 [9.0–12.0]	10.0 [8.0–11.0]	11.0 [10.0–12.5]	11.0 [10.0–12.0]	.26
Identification	10.0 [7.5–11.0]	10.0 [7.0–11.0]	11.0 [9.5–12.5]	9.0 [9.0–10.0]	.26
TDI score, median [P25‐P75]	23.5 [20.5–28.4]	22.8 [18.5–27.0]	27.0 [23.5–28.1]	23.3 [20.5–28.3]	.35
Anosmic, *n* (%)	7 (15.9%)	4 (21.1%)	2 (12.5%)	1 (11.1%)	
Hyposmic, *n* (%)	37 (84.1%)	15 (78.9%)	14 (87.5%)	8 (88.9%)	.75
VAS smell, median [P25‐P75]	4.0 [1.0–6.0]	2.5 [0.8–4.0]	5.0 [2.0–7.0]	3.0 [1.8–6.3]	.35
SNOT‐22, median [P25‐P75]	22.0 [12.0–38.5]	24.5 [10.0–41.8]	18.0 [15.0–26.0]	32.0 [14.5–60]	.63
Parosmia, no (%)	31 (70.5%)	16 (84.2%)	7 (43.8%)	8 (89.9%)	.01*
Findings at second assessment (T_1_)					
Sniffin' Sticks, median [P25‐P75]					
Threshold	5.5 [3.3–6.6]	5.0 [2.6–5.8]	5.6 [4.0–7.4]	5.5 [3.8–7.5]	.52
Discrimination	11.0 [10.0–13.0]	11.0 [9.5–12.0]	11.0 [10.0–13.3]	12.0 [11.0–13.0]	.33
Identification	10.5 [9.8–12.0]	10.0 [9.0–12.0]	11.5 [10.0–12.0]	10.0 [10.0–10.0]	.28
TDI score, median [P25‐P75]	26.6 [23.0–30.0]	24.8 [22.6–28.8]	27.5 [24.8–32.7]	29.5 [24.5–30.8]	.27
Anosmic, *n* (%)	0 (0.0%)	0 (0.0%)	0 (0.0%)	0 (0.0%)	
Hyposmic, *n* (%)	33 (75.0%)	16 (84.2%)	11 (68.7%)	6 (66.7%)	.47
Normosmic, *n* (%)	11 (25.0%)	3 (15.8%)	5 (31.3%)	3 (33.3%)	
VAS smell, median [P25‐P75]	5.0 [3.0–7.0]	5 [3.0–6.0]	5.8 [4.8–8.0]	5.0 [2.0–8.0]	.27
SNOT‐22, median [P25‐P75]	18.0 [8.8–26.0]	21.0 [10.5–27.5]	17.0 [9.8–23.0]	8.0 [5.0–26.0]	.32
Parosmia, no (%)	27 (61.4%)	11 (57.9%)	8 (50.0%)	8 (88.9%)	.35

Abbreviations: SNOT‐22, SinoNasal Outcome Test‐22 items; TDI, threshold + discrimination + identification; VAS, visual analogue scale.

* Significant *p*‐values. Level of significance *p* < .05.

### 3.4 Effects of the therapy on olfaction

A statistically significant improvement in the TDI score was demonstrated at follow‐up in patients receiving the combined treatment (*p* = .01) and those doing OT alone (*p* = .04) whereas a significant improvement in VAS score was shown only for patients in the former group (*p* = .01). No significant changes were noted in group C or in the SNOT‐22 score or in the number of parosmics for all groups (Table 3). In six patients (31.6%) in group A, the TDI improvement was above the MCID of 5.5 points in TDI score^22^ when compared to five patients (31.3%) in group B and four patients (44.4%) in group C. No significant differences were observed when comparing the number of patients reaching the MCID improvement in the three groups (*p* = .78).

**TABLE 3:** Changes between T_0_ and T_1_ for the available variables and statistical differences

	Patients with OD (*n* = 44)	Group A_S+OT_ (*n* = 19)	Group B_OT_ (*n* = 16)	Group C_None_ (*n* = 9)	*p*‐value_A‐B‐C_
Sniffin' Sticks, median [IQR]					
Threshold	+1.25 [3.75] (*p* = .004*)	+0.50 [2.62] (*p* = .11)	+2.00 [3.88] (*p* = .06)	+2.75 [4.75] (*p* = .23)	.58
Discrimination	+1.00 [3.00] (*p* = .008*)	+1.00 [3.00] (*p* = .06)	+1.00[3.50] (*p* = .17)	+2.00 [5.00] (*p* = .23)	.94
Identification	+1.00 [3.50] (*p* = .01*)	+0.00 [3.50] (*p* = .09)	+0.00 [3.50] (*p* = .27)	+1.00 [1.00] (*p* = .65)	.85
TDI score, median	+2.25 [8.25] (*p* = .0003*)	+2.25 [5.75] (*p* = .01*)	+2.5 [9.38] (*p* = .04*)	+0.75 [9.75] (*p* = .12)	.99
VAS smell, median [IQR]	+2.00 [3.00] (*p* = .003*)	+2.00 [2.62] (*p* = .01*)	+3.00 [5.00] (*p* = .22)	+1.00 [2.25] (*p* = .09)	.84
SNOT‐22, median [IQR]	−1.00 [13.00] (*p* = .59)	−1.50 [12.00] (*p* = .57)	0.00 [14.00] (*p* = .89)	−8.00 [28.00] (*p* = .62)	.77
Parosmia, no (%)	−4 (0.09%) (*p* = .46)	−5 (0.26%) (*p* = .51)	+1 (0.06%) (*p* = 1)	0 (0.0%)	.06

*Note*: The sign “+” shows an increase in the recorded values whereas the sign “−” highlights a decrease. Please note that values represent changes either in the median values (Sniffin' Sticks, VAS smell, SNOT‐22) or number of observations (Parosmia).

Abbreviations: SNOT‐22, SinoNasal Outcome Test‐22 items; TDI, threshold + discrimination + identification; VAS, visual analogue scale.

* Significant *p*‐values. Level of significance *p* < .05.

### 3.5 Influence of available variables on smell improvement

Presence of comorbidities negatively influenced the TDI and identification scores in group A (*p* = .04 and *p* = .03 respectively) and the discrimination and identification scores in group B (*p* < .001 and *p* = .007 respectively). Age and sex (male) negatively influenced identification score in group B only (*p* < .001 for both) whereas the length of OD negatively influenced threshold and discrimination scores in group A (*p* = .02 and *p* = .01 respectively) and the discrimination and identification scores in group B (*p* < .001 and *p* = .004 respectively) (Table 4). All the other variables were found to not influence smell recovery.

**TABLE 4:** Influence of the available variables on smell recovery for Group A and Group B

	Group A_S+OT_				Group B_OT_			
	TDI	Threshold	Discrimination	Identification	TDI	Threshold	Discrimination	Identification
Age (≤50 years)	‐	‐	‐	‐	‐	‐	‐	<0.001*
Sex (male)	‐	0.29	‐	‐	‐	‐	0.06	<0.001*
Comorbidities	0.04*	‐	0.06	0.03*	‐	‐	<0.001*	0.007*
Smoking (yes)	‐	0.10	0.009*	‐	‐	‐	0.14	0.28
Previous oral steroid	‐	0.04*	‐	‐	‐	‐	‐	‐
Previous nasal steroid	‐	0.25	‐	‐	‐	‐	0.002*	0.002*
Previous multivitamins	‐	0.05	‐	‐	‐	‐	<0.001*	<0.001*
Previous OT	‐	0.04*	‐	‐	‐	‐	‐	‐
Length of OD (≤300 days)	‐	0.06	0.17	‐	0.02*	‐	<0.001*	0.004*

*Note*: Please note that not all the variables enter the multiple regression model but only those found to be significant at the stepwise selection based on AIC.

Abbreviations: SNOT‐22, SinoNasal Outcome Test‐22 items; TDI, threshold + discrimination + identification; VAS, visual analogue scale.

* Significant *p*‐values. Level of significance *p* < .05.

## 4 DISCUSSION

Corticosteroids have been considered as a therapeutic option for PIOD with many studies showing promising results.^23^, ^24^, ^25^, ^26^, ^27^ It has been hypothesized that some patients with persistent PIOD may have an undetectable (not macroscopically evident) ongoing inflammation in the olfactory neuroepithelium^28^, ^29^, ^30^ which could explain why some people could respond better than others to steroidal treatment.^30^, ^31^ However, in the absence of large randomized‐controlled trials, evidence supporting its use in PIOD remains weak. So far, a unanimous consensus has not been reached and clear guidelines do not exist. In January 2021, an experts panel concluded that “oral and topical steroids may still have a role in the management” of PIOD and “may be used in carefully selected patients”^15^ while in another international consensus issued a month later on the treatment of COVID‐19‐related OD the majority of the authors thought that “systemic CCS should not be considered as standard‐of‐care” although these could “have a potential place” in its treatment.^32^

Our results failed to demonstrate a clear superiority of taking corticosteroids in combination with OT over OT alone. In fact, both treatments were found to improve TDI score at follow‐up although none was superior to the other (*p* = .99). Nevertheless, a higher statistically significant improvement was demonstrated in the group of patients taking the combined treatment (*p* = .01 vs. *p* = .04). When looking at the MCID for the TDI score for single patient in each group, we observed a very similar percentage of patients who reached the MCID in the two treatment groups (31.6% in group A vs. 31.3% in group B) with a slightly higher number of patients in group C, although this was not statistically significant (*p* = .78). Nonetheless, a statistically significant improvement of the VAS score (*p* = .01) was observed only in those having the combined treatment. The lack of statistically significant differences of baseline characteristics between the three groups, helped us to rule out any selection bias in treatment choice. Overall, these results seem to suggest a benefit, at least in the reported OD, of adding a short course of corticosteroids to OT in the management of COVID‐19‐related OD. In this regard, our data corroborate previous findings by Le Bon et al.^25^ who found that only patients with combined therapy (10‐day course of 32 mg of methylprednisolone once daily combined with OT) significantly improved olfactory function when compared to those who did the OT alone. However, our patients had a considerably longer length of OD (7.5 months on average) compared to Le Bon et al. subjects (5 weeks on average). A recent systematic review by Yuan et al.^26^ concluded that “a combination of steroids and OT is more efficient than OT only in managing OD from post‐viral OD.” In 2018, Nguyen and Patel^27^ found that steroid irrigation (Budesonide respules in a 0.5‐mg/2‐ml dose) in combination with OT was superior to OT alone in improving olfactory function in patients with anosmia of different causes (46.6% were PIOD). In a retrospective study conducted on 46 adults, Fleiner et al.^8^ concluded that OT with a topical nasal steroid (not better described) was more effective than OT alone, especially in the subgroup of patients with PIOD. It must be stated that, in addition to the way of administration, corticosteroid molecules differ in terms of their anti‐inflammatory potencies and duration of action^33^ which could eventually influence their potential effect to improve sense of smell. However, to our knowledge, the best corticosteroid molecule to use in COVID‐19‐related OD, or broadly in post‐viral OD, has not yet been identified.

Today, most of the authors agree that, considering the systemic side effects of taking oral corticosteroids, it is not recommended to use them more than 2 weeks for the treatment of COVID‐19‐related OD.^34^ As an option, giving a short course of oral steroids for 3–4 days has been suggested as a diagnostic tool,^31^ followed then by a full course of steroids completing 2 weeks for those responding. However, this would require an extra follow‐up to assess treatment response which could not always be feasible in the context of a stretched national health system.

A strong association between the time of initiation of corticosteroids therapy and smell recovery rate has been confirmed in patients with PIOD. Experts agree that oral corticosteroids could have a role only if administered in the early stage of COVID‐19‐related OD^15^ event though the overall consensus is to not suggest them within the first 3 weeks after OD onset due to the high rate of spontaneous recovery.^2^, ^17^, ^32^ However, the question remains whether it is worthwhile trying oral corticosteroids in patients with a persistent OD (longer than 6 months). In this regard, Genetzaki et al.^31^ noted a smell improvement also in patients with persistent OD (up to 12 months) receiving oral corticosteroids plus OT. In our study, a significant improvement of the TDI score was observed in group A with patients having an average length of OD of 7.1 months. However, the length of OD did not influence smell recovery in group A whereas an effect was noted in group B on TDI, threshold, and identification scores with a cut‐off of 300 days found to be significant for all the three scores. This suggests that an early initiation of the OT (before 10 months) could give a better benefit in terms of olfactory improvement. Interestingly, the lack of influence of the time variable on the olfactory recovery of patients taking the combined treatment would indicate its effectiveness irrespective of the length of OD.

We also found that patients in both groups who had had previous treatments for OD responded better to the therapy in terms of olfactory scores at follow‐up. Similarly, the presence of comorbidities significantly correlated with smell recovery in both treatment groups whereas an impact of age (younger than 50 years) and sex (male) was found to influence identification scores only in those who did the OT alone, as previously noted.^2^

The decision over the best way of administering corticosteroids (oral vs. topical vs. combination) still remains a matter of debate. Despite some studies seem to show no benefit of topical steroid in improving PIOD,^35^, ^36^, ^37^ delivery method could influence response to treatment. The majority of the authors agree that nasal corticosteroids sprays are not useful because they cannot reach the olfactory clefts. On the other side, rinsing with a topical steroid irrigation^27^ or delivering steroid drops in the Kaiteki position^38^ has been reported to be helpful. Given the potential benefits of intranasal steroid drops, we offered a combined treatment of oral and topical steroids for a total length of treatment of 4 weeks.

Finally, our data also highlight the role of OT in persistent PIOD, as demonstrated by the fact that no statistically significant improvement was observed in those who did not do it (group C).

### 4.1 Strengths and limitations

This study is the first one looking at the role of corticosteroids in patients with a persistent COVID‐19‐related OD. Also, all patients considered in the study had no signs of paranasal inflammation, as demonstrated by a clear MRI head. This allowed us to be more confident that any smell improvement observed in the steroid group would have not been confounded by treating an underlying sinonasal disease. The main limitation of the study is its non‐randomized non‐blinded design as treatments suggested were not randomly assigned. However, this represents a real‐life study and it was not initially designed as a prospective controlled trial. Group C did not reach the minimal sample size; therefore, we cannot exclude that the results observed regarding this group were affected by a casual effect. Even though it could be considered a controlled study for the presence of two different control groups, their inclusion was not part of the initial study design but was a consequence of patients' own choice to take or not the treatment suggested. As an additional consequence of that, the patients reported outcomes (i.e., VAS and SNOT‐22) might have been biased whereas those receiving the combined treatment were more prone to believe they could have achieved an improvement at the end of the treatment. Also, by giving a combination of oral and topical steroid drops to patients in group A, we were not able to conclude whether the observed smell improvement was due to a particular formulation of corticosteroids or to the combination of both.

## 5 CONCLUSIONS

Our study confirms the importance of OT in the treatment of persistent COVID‐19‐related OD suggesting that the addition of corticosteroids may also give a benefit in terms of patient's perceived olfaction. Topical steroid drops administered in the Kaiteki position may contribute to oral corticosteroids effect by targeting directly the olfactory neuroepithelium. However, benefits of corticosteroids must be considered against their systemic side effects and randomized controlled studies on bigger populations are strongly encouraged to better clarify their role in the treatment of persistent PIOD.

## CONFLICT OF INTEREST

The authors declare that they have no conflict of interest.
